# Autoimmune Encephalitis in Later Life: A Missed Opportunity in Routine Geriatric Care

**DOI:** 10.7759/cureus.104404

**Published:** 2026-02-27

**Authors:** Syamasis Bandyopadhyay, Arghya Sahu, Sandip K Chandra, Aheli Ghosh Dastidar, Sourav Pratihar

**Affiliations:** 1 Internal Medicine, Apollo Hospitals, Kolkata, IND; 2 Medicine, Apollo Hospitals, Kolkata, IND

**Keywords:** autoimmune encephalitis, delirium, older adults, plasma exchange, positron-emission tomography

## Abstract

Autoimmune encephalitis (AE) is an increasingly recognized but frequently overlooked cause of subacute cognitive and behavioral decline in older adults, in whom delirium is typically attributed to metabolic, infectious, or vascular disorders. We describe an elderly man who presented with persistent encephalopathy following correction of hyponatremia. Despite normal structural neuroimaging and negative cerebrospinal fluid autoimmune markers, he remained delirious. Electroencephalography revealed diffuse slowing, and fluorodeoxyglucose positron emission tomography demonstrated characteristic hypermetabolic activity involving the temporal lobes, basal ganglia, thalami, and cerebellum, strongly suggestive of AE. Initiation of high-dose corticosteroids followed by therapeutic plasma exchange resulted in marked clinical improvement. This case highlights the diagnostic complexities of AE in geriatric practice, particularly the limitations of conventional testing and the value of clinical suspicion backed by functional neuroimaging. Greater clinical vigilance is required to avoid delayed diagnosis and treatment, which may substantially improve outcomes even in severe presentations.

## Introduction

Delirium and encephalopathy are among the most common neurologic syndromes encountered in hospitalized older adults and are most frequently attributed to infection, metabolic disturbances, dehydration, medication effects, or vascular events [1]. Delirium, as defined by Diagnostic and Statistical Manual of Mental Disorders, Fifth Edition, Text Revision (DSM-5-TR), refers to an acute and fluctuating disturbance in attention and awareness developing over hours to days, whereas encephalopathy describes diffuse cerebral dysfunction resulting from metabolic, toxic, structural, infectious, or immune-mediated causes. Subacute encephalopathy typically evolves over days to weeks and may manifest with progressive cognitive decline, behavioral change, or altered consciousness.

Autoimmune encephalitis (AE) represents an immune-mediated inflammatory disorder of the central nervous system characterized by subacute cognitive dysfunction, psychiatric symptoms, seizures, or movement abnormalities [2,3]. Although increasingly recognized, AE remains uncommon compared with metabolic or infectious causes of encephalopathy. Population-based studies from high-income countries estimate the annual incidence at approximately 0.8-1.5 per 100,000 persons, with lower representation among older adults compared with younger cohorts [4]. Data from low- and middle-income settings remain limited, but hospital-based series suggest that AE accounts for a small proportion of encephalopathy cases in geriatric practice. Despite its rarity, the condition is clinically significant because it is potentially reversible.

In older adults, presentations are often atypical and may lack seizures or prominent psychiatric features, making differentiation from delirium or early neurodegenerative disease challenging [5,6]. Up to 50% of clinically probable cases are seronegative [7], and structural magnetic resonance imaging may be normal or nonspecific, particularly in individuals with age-related small-vessel changes [8]. Functional imaging, including fluorodeoxyglucose positron emission tomography, may demonstrate metabolic abnormalities even when structural imaging is unrevealing [8,9]. We present a case of probable antibody-negative AE in an older adult, highlighting the importance of structured diagnostic reasoning in a condition that is rare yet potentially reversible.

## Case presentation

A man in his late 70s, previously independent and cognitively intact, developed progressive confusion, reduced interaction, daytime somnolence, and agitation over three weeks. The illness began with impaired attention and sleep disturbance and progressed to disorientation and fluctuating responsiveness. There was no prior history of dementia, psychiatric illness, autoimmune disease, malignancy, toxin exposure, or recent vaccination. His medical history included well-controlled hypertension managed with amlodipine.

During week two of illness, he was admitted elsewhere with profound hyponatremia of 106 mmol/L. Evaluation demonstrated hypotonic hyponatremia. Serum osmolality was reduced, and urine studies, including urine osmolality and urine sodium, were obtained, and those were unremarkable. Clinical examination demonstrated mild hypovolemia with dry mucous membranes and reduced skin turgor. Thyroid function and adrenal function were normal. Sodium improved with cautious administration of hypertonic saline and volume repletion. Correction was carefully monitored and did not exceed 8 mmol/L per 24 hours. Serial measurements confirmed gradual normalization. Despite biochemical correction, cognitive impairment persisted.

During week three, he was transferred for further evaluation. On admission, he was afebrile and hemodynamically stable. Neurological examination revealed global disorientation with inconsistent response to commands and a Glasgow coma scale score of 10/15. Cranial nerves were intact. Motor examination demonstrated generalized rigidity and symmetric weakness without focal deficits. Deep tendon reflexes were preserved.

Laboratory testing showed sodium 122 mmol/L, potassium 2.4 mmol/L, creatinine 1.4 mg/dL, and blood urea nitrogen 28 mg/dL. Serum ammonia, thyroid-stimulating hormone, vitamin B12, and lactate were within reference ranges. C-reactive protein was mildly elevated. Chest radiography showed right lower lobe consolidation consistent with aspiration pneumonia. He was treated with ceftriaxone and azithromycin. Neurological symptoms had preceded antibiotic therapy and did not worsen with treatment. Electrolytes and inflammatory markers normalized, yet mental status remained impaired (Table 1).

**Table 1:** Laboratory, cerebrospinal fluid, and imaging findings used to exclude alternative diagnoses and support antibody-negative autoimmune encephalitis, including inflammatory CSF changes, hyponatremia, negative neuronal antibodies, and clinical response to immunotherapy ANA: Antinuclear antibody; ANCA: Antineutrophil cytoplasmic antibody; CRP: C-reactive protein; CSF: Cerebrospinal fluid; EEG: Electroencephalography; FDG PET-CT: Fluorodeoxyglucose positron emission tomography–computed tomography; HSV: Herpes simplex virus; MRI: Magnetic resonance imaging; PCR: Polymerase chain reaction; VZV: Varicella zoster virus.

Investigation	Result (Reference Range)	Interpretation
Sodium	122 mmol/L (135–145 mmol/L)	Hyponatremia
Potassium	2.4 mmol/L (3.5–5.0 mmol/L)	Hypokalemia
Creatinine	1.4 mg/dL (0.7–1.3 mg/dL)	Mild renal impairment
Blood urea nitrogen	28 mg/dL (7–20 mg/dL)	Mild azotemia
Serum ammonia	32 µg/dL (15–45 µg/dL)	Within reference range
Vitamin B12	482 pg/mL (200–900 pg/mL)	Within reference range
Thyroid-stimulating hormone	2.1 mIU/L (0.4–4.0 mIU/L)	Within reference range
C-reactive protein	1.8 mg/dL (<0.5 mg/dL)	Mild elevation
Antinuclear antibody	Negative (Negative)	No serologic evidence of connective tissue disease
Antineutrophil cytoplasmic antibody	Negative (Negative)	No serologic evidence of ANCA-associated vasculitis
Human immunodeficiency virus serology	Negative (Negative)	HIV infection excluded
Rapid plasma reagin	Nonreactive (Nonreactive)	Syphilis excluded
Magnetic resonance imaging brain	Chronic small-vessel ischemic changes; no acute diffusion restriction or limbic signal abnormality	No acute structural lesion identified
EEG	Diffuse 5–6 Hz background slowing; intermittent temporal sharp waves (normal posterior dominant rhythm 8–12 Hz)	Diffuse cerebral dysfunction with focal temporal cortical irritability
Cerebrospinal fluid protein	92 mg/dL (15–45 mg/dL)	Elevated
Cerebrospinal fluid cell count	5 cells/mm³ (0–5 cells/mm³)	Upper limit of normal
Cerebrospinal fluid glucose	68 mg/dL (45–80 mg/dL)	Within reference range
Cerebrospinal fluid PCR (HSV and VZV)	Negative (Negative)	No virologic evidence of HSV or VZV infection
Blood and cerebrospinal fluid cultures	Sterile (Sterile)	No bacterial growth
Neuronal antibody panel (serum and CSF): N-methyl-D-aspartate receptor, leucine-rich glioma-inactivated 1, contactin-associated protein-like 2, α-amino-3-hydroxy-5-methyl-4-isoxazolepropionic acid receptor, γ-aminobutyric acid type B receptor, and dipeptidyl-peptidase-like protein 6	Negative (Negative)	No detectable neuronal surface antibodies
Paraneoplastic antibody panel: Hu, Yo, Ma2, CV2/CRMP5, amphiphysin, glutamic acid decarboxylase 65	Negative (Negative)	No detectable paraneoplastic antibodies
Whole-body FDG PET-CT	No hypermetabolic extracranial lesion	No metabolically active systemic malignancy identified
FDG PET-CT brain	Bilateral temporal hypermetabolism with basal ganglia and thalamic involvement	Abnormal metabolic pattern
Repeat EEG	Restoration of organized posterior dominant rhythm	Improvement in background activity

**Figure 1:**
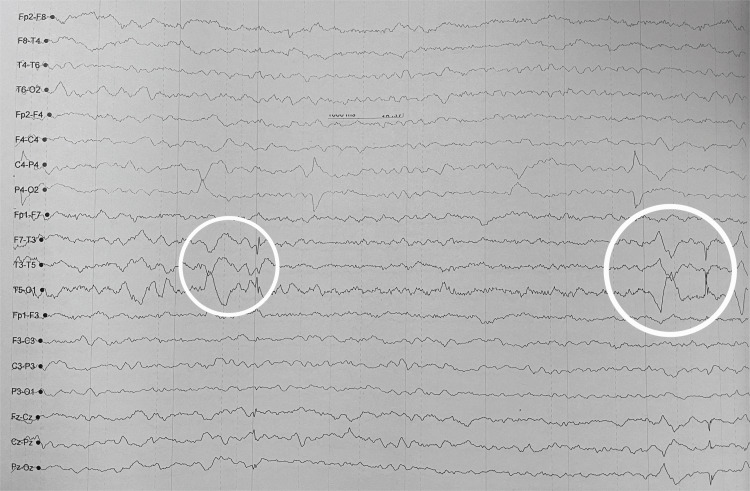
Electroencephalography demonstrating diffuse background slowing with intermittent temporal sharp waves and spike–slow wave complexes, most prominent over the left temporal region (F7–T3–T5 derivations; circled). The findings are consistent with diffuse encephalopathy and focal temporal cortical irritability, supporting limbic involvement in autoimmune encephalitis.

Magnetic resonance imaging of the brain demonstrated chronic small-vessel ischemic changes and an old lacunar infarct without acute diffusion restriction, hemorrhage, or limbic signal abnormalities. No imaging features were consistent with osmotic demyelination. Electroencephalography revealed diffuse background slowing at 5-6 Hz with intermittent temporal sharp waves, indicating diffuse cerebral dysfunction with focal temporal cortical irritability (Figure 1).

Cerebrospinal fluid analysis demonstrated elevated protein of 92 mg/dL with 5 cells/mm³ and normal glucose. Gram stain, bacterial cultures, tuberculosis testing, and viral polymerase chain reaction assays, including herpes simplex virus and varicella zoster virus, were negative. HIV serology and rapid plasma reagin testing were negative (Table 1).

Serum and cerebrospinal fluid neuronal antibody testing for N-methyl-D-aspartate receptor, leucine-rich glioma-inactivated 1, contactin-associated protein-like 2, AMPA receptor, GABA B receptor, and DPPX antibodies were negative. Paraneoplastic antibodies, including Hu, Yo, Ma2, CV2, CRMP5, amphiphysin, and GAD65, were negative (Table 1).

**Figure 2:**
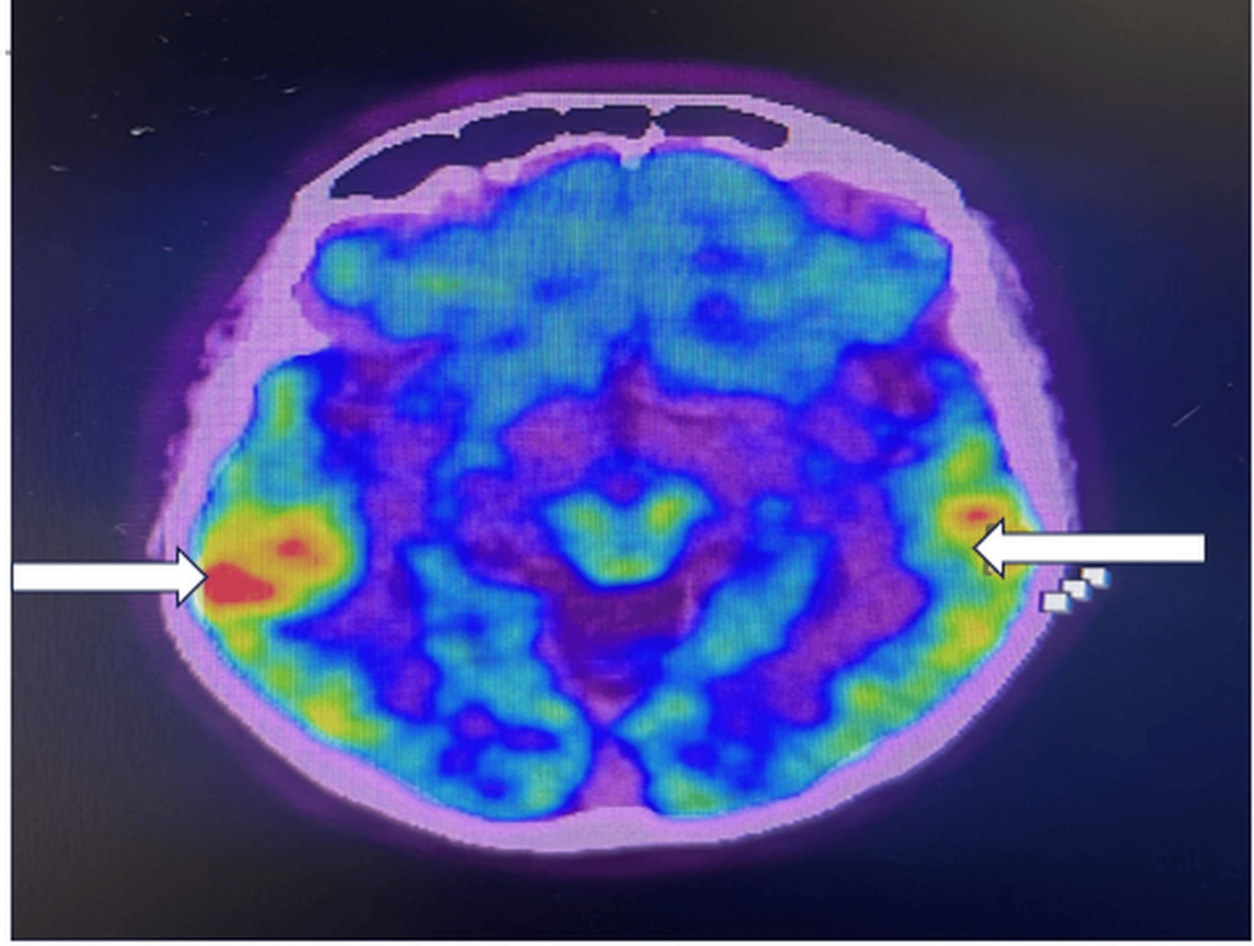
Axial FDG PET-CT brain image showing bilateral temporal lobe hypermetabolism (arrows), more prominent on the right, consistent with limbic involvement in autoimmune encephalitis FDG PET-CT: Fluorodeoxyglucose positron emission tomography–computed tomography.

Because structural imaging did not explain the severity of encephalopathy, FDG PET-CT brain imaging was obtained and demonstrated bilateral temporal hypermetabolism with additional involvement of basal ganglia and thalami without any evidence of active malignant disease in the rest of the body (Figure 2). These findings were supportive of an inflammatory or autoimmune process but were not pathognomonic.

The diagnostic reasoning followed established criteria for probable antibody-negative AE as proposed by Graus et al. [2]. The patient had a subacute onset of cognitive dysfunction over less than three months, supportive electroencephalographic abnormalities, inflammatory cerebrospinal fluid findings, reasonable exclusion of alternative diagnoses, including metabolic, infectious, toxic, vascular, demyelinating, and paraneoplastic causes, and subsequent response to immunotherapy.

High-dose intravenous methylprednisolone at 1 g daily for five days was initiated. Partial improvement occurred, but significant cognitive impairment persisted. Plasma exchange was commenced with five exchanges over 10 days. Plasma exchange was selected due to availability, rapid removal of pathogenic antibodies, and incomplete response to corticosteroids. Intravenous immunoglobulin was not used initially due to institutional resource considerations and protocol preference for plasma exchange in severe presentations.

After the third exchange, a marked improvement in orientation, attention, and motor function was observed. Repeat electroencephalography showed restoration of an organized background rhythm. He was discharged on a tapering corticosteroid regimen. At the six-week follow-up, he remained cognitively improved and ambulatory. Longitudinal monitoring was arranged, given evidence that a proportion of patients may experience residual cognitive decline despite initial recovery.

## Discussion

This case highlights the diagnostic challenge of persistent subacute encephalopathy in older adults and underscores the importance of avoiding diagnostic anchoring. Hyponatremia is a well-recognized cause of delirium in elderly patients [1]; however, in this case, sodium correction was performed cautiously within recommended safety limits, and no imaging findings were consistent with osmotic demyelination. The persistence of cognitive dysfunction despite biochemical normalization argued against a purely metabolic cause.

Alternative diagnoses were systematically excluded. Infectious etiologies were ruled out through negative cerebrospinal fluid cultures and viral polymerase chain reaction testing. Antibiotic-associated neurotoxicity was unlikely, given the timing of neurologic symptoms and the agents used. Vascular dementia was considered but deemed improbable due to the absence of stepwise decline, new infarcts, or focal deficits. Comprehensive paraneoplastic screening, including antibody testing and whole-body imaging, revealed no evidence of malignancy.

The diagnosis was approached deductively using the criteria proposed by Graus et al. for probable antibody-negative AE [2]. The patient met criteria based on subacute onset, supportive electroencephalographic abnormalities, inflammatory cerebrospinal fluid findings, and exclusion of alternative causes. Although neuronal antibody testing was negative, seronegativity does not exclude AE, as up to 50% of clinically probable cases lack detectable antibodies [7].

Structural magnetic resonance imaging may be normal in antibody-negative disease, particularly in older adults with background small-vessel changes [8]. Electroencephalography findings were supportive but nonspecific. FDG PET demonstrated bilateral temporal hypermetabolism, a pattern described in AE when structural imaging is nondiagnostic [8,9]. These findings were supportive rather than diagnostic in isolation and were interpreted within the broader clinical context.

AE remains rare compared with common causes of delirium such as infection, metabolic disturbance, or medication effects [1,4]. Nevertheless, its potential reversibility warrants consideration in cases of persistent subacute encephalopathy. Early immunotherapy is recommended once alternative causes are reasonably excluded [3,10]. In this patient, partial response to corticosteroids followed by more substantial improvement after plasma exchange supports an immune-mediated process, although causality cannot be definitively established in a single case.

Overall, this case emphasizes that while AE is uncommon in older adults, it should remain in the differential diagnosis when encephalopathy persists despite correction of metabolic or infectious triggers. A structured, deductive approach integrating clinical course, exclusion of alternatives, electroencephalography, cerebrospinal fluid analysis, and functional imaging can facilitate timely recognition without overstating diagnostic certainty.

## Conclusions

AE is an uncommon but potentially reversible cause of subacute encephalopathy in older adults. In this case, persistent cognitive dysfunction despite cautious correction of hyponatremia and appropriate treatment of infection prompted a structured reassessment. Systematic exclusion of metabolic, infectious, vascular, toxic, and paraneoplastic causes, combined with supportive electroencephalographic, cerebrospinal fluid, and functional imaging findings, fulfilled the established criteria for probable antibody-negative AE.

Although clinical improvement followed immunotherapy, causality cannot be definitively established in a single case. Structural imaging and serologic testing may be nondiagnostic in seronegative disease, and ancillary studies must be interpreted cautiously within the broader clinical context. This report emphasizes the importance of maintaining diagnostic flexibility in older adults with persistent subacute encephalopathy. A careful, deductive approach may identify rare but treatable immune-mediated conditions without overstating their frequency in routine geriatric practice.
